# Nucleic acids and proteins carried by exosomes from various sources: Potential role in liver diseases

**DOI:** 10.3389/fphys.2022.957036

**Published:** 2022-09-23

**Authors:** Danna Xie, Baolin Qian, Xun Li

**Affiliations:** ^1^ The First Clinical Medical College of Lanzhou University, Lanzhou, China; ^2^ Department of Hepatic Surgery, The First Affiliated Hospital of Harbin Medical University, Harbin, China; ^3^ Department of General Surgery, the First Hospital of Lanzhou University, Lanzhou, China; ^4^ Key Laboratory of Biotherapy and Regenerative Medicine of Gansu Province, Lanzhou, China; ^5^ Center for Cancer Prevention and Treatment, School of Medicine, Lanzhou University, Lanzhou, China; ^6^ Gansu Provincial Institute of Hepatobiliary and Pancreatic Surgery, Lanzhou, China

**Keywords:** liver disease, exosomes, nucleic acid, protein, biomarker

## Abstract

Exosomes are extracellular membrane-encapsulated vesicles that are released into the extracellular space or biological fluids by many cell types through exocytosis. As a newly identified form of intercellular signal communication, exosomes mediate various pathological and physiological processes by exchanging various active substances between cells. The incidence and mortality of liver diseases is increasing worldwide. Therefore, we reviewed recent studies evaluating the role of exosomes from various sources in the diagnosis and treatment of liver diseases.

## Introduction

Liver diseases have a high incidence and mortality worldwide, accounting for approximately 3.5% of all global deaths annually (Byass, 2014; Asrani et al., 2019; Xiao et al., 2019a). In China, approximately 300 million people suffer from liver diseases. Liver diseases also affect 30 million people each year in the United States. In the European Union, 29 million people are affected by liver diseases (Ma et al., 2019). Liver diseases comprise drug-induced liver injury (DILI), hepatic ischemia reperfusion injury (HIRI), hepatic fibrosis (HF), liver failure (LF) and liver cancer, among others (Llovet et al., 2021). DILI is a common and serious adverse drug reaction that can lead to acute liver failure (ALF) and death (Katarey and Verma, 2016; Shen et al., 2019). Pathological types of DILI include inflammatory necrosis, cholestasis, steatosis and steatohepatitis, vascular injury, and mild lesion types (Gasmi and Kleiner, 2020). HIRI is the main cause of liver dysfunction and LF after transplantation (Ingram et al., 2022) and can lead to hepatocyte necrosis and distant organ damage (Hua et al., 2019), which are associated with significant mortality (Cornide-Petronio et al., 2020). HF is the ultimate common pathway for chronic or persistent liver injury and often progresses to life-threatening liver cirrhosis and liver cancer in advanced stages. Mass deposition of extracellular matrix (ECM) is an important feature of HF, with destruction of the normal structure and function of the liver (Aydin and Akcali, 2018). LF is characterized by heptatocytic injury and decreased synthetic function and may be caused by a range of factors (Arshad et al., 2020). Histological analysis of LF typically demonstrates new or old necrotic lesions (Dong et al., 2020). Mortality from liver cancer ranks third in the world for cancer deaths (Sung et al., 2021). The histological types of primary liver cancer are divided into hepatocellular, bile duct epithelial, and mixed types, of which hepatocellular carcinoma is the most common (accounting for more than 90%) (Villanueva, 2019). Without early intervention, liver diseases may rapidly progress and have a dismal prognosis (Asrani et al., 2019). Therefore, there is a clinical need for novel biomarkers related to liver diseases.

There is increasing scientific interest in the role of exosomes in human disease. Exosomes are a specialized type of extracellular vesicle with a diameter of 30–150 nm (Turturici et al., 2014; Yanez-Mo et al., 2015; Tkach and Thery, 2016). Exosomes observed by transmission electron microscopy are typically disc-shaped or hemispherical with a concave surface. Exosomes are formed by cells through the process of “endocytosis-fusion-discharge” (Corrado et al., 2013). Exosomes have been shown to be present in plasma, urine, saliva, and ascites (Hu et al., 2021; Wen et al., 2021; Nafar et al., 2022). A range of cell types secrete exosomes, including tumor, dendritic, and stem cells (Wu et al., 2021a; Wu et al., 2021b; Lyu et al., 2021; Shao et al., 2021). Various surface molecules of exosomes can directly activate cell receptors and are involved in the exchange of substances between cells (Jones et al., 2018; Yu et al., 2021). Furthermore, exosomes can participate in intercellular signal transduction by carrying proteins, nucleic acids, lipids, and other signaling molecules (Eldh et al., 2010; Yu et al., 2021; Osawa et al., 2022).

**FIGURE 1:**
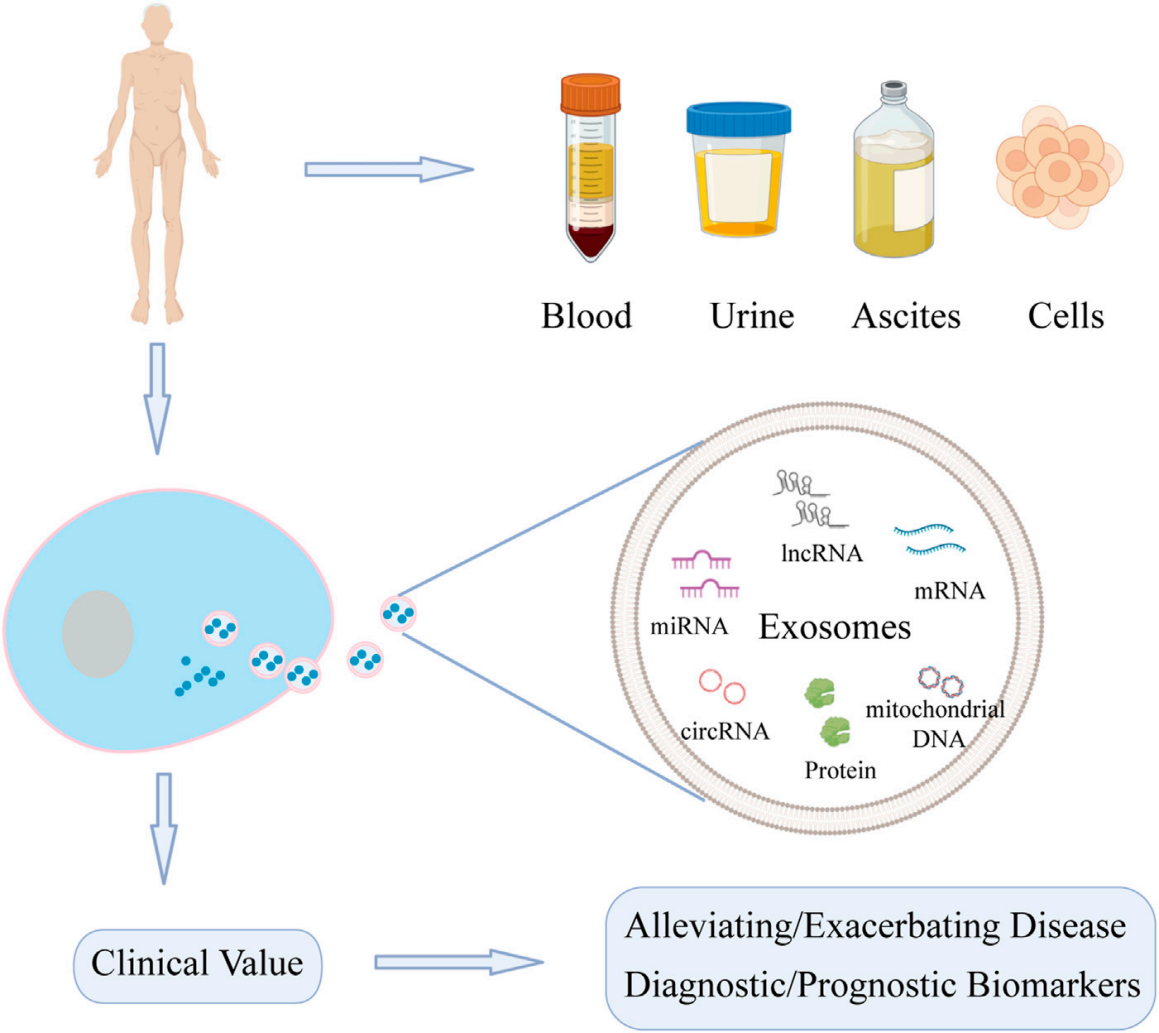
Function of nucleic acids and proteins carried by exosomes from different sources on liver diseases. Exosomes can be extracted from various sources. Nucleic acids and proteins carried by exosomes can not only act as biomarkers for diagnosis and treatment, but also may alleviate or exacerbate liver diseases.

The liposome membrane of exosomes can prevent degradation of carried contents, of which nucleic acids and proteins are key mediators of downstream functions (Koga et al., 2011; Wang et al., 2021a; Zhao et al., 2021a). Increasing number of studies have shown that nucleic acids and proteins carried by exosomes are involved in the pathogenesis and progression of liver diseases, including roles in tumor growth, cell migration, fibrosis, and regeneration of hepatocytes (Wu et al., 2018; Jiao et al., 2021a; Jiao et al., 2021b; Shi et al., 2021). Exosomes may have potential as biomarkers in liver diseases. Thus, this article summarizes the role of nucleic acids and proteins carried by exosomes in liver diseases (DILI, LIRI, HF, LF, and liver cancer) (Figure 1).

## Exosomes and drug-induced liver injury

**FIGURE 2:**
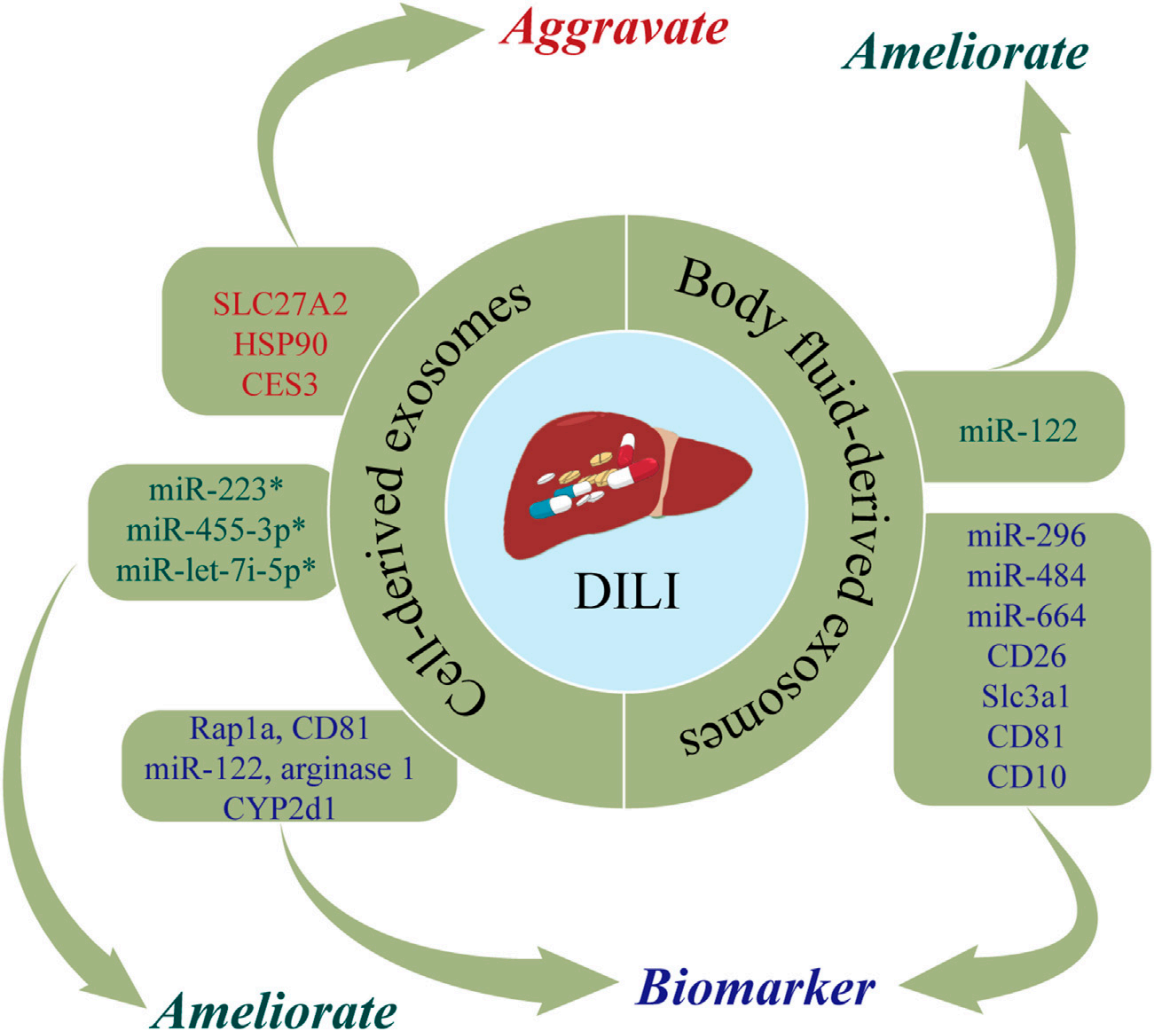
The roles of nucleic acids and proteins carried by exosomes in drug-induced liver injury (DILI). Nucleic acids or proteins that may aggravate DILI are highlighted in red, ameliorating DILI are highlighted in green, and biomarkers are highlighted in purple. * refers to nucleic acids and proteins carried by exosomes from mesenchymal stem cell.

DILI is defined as liver dysfunction induced by drugs or metabolites and characterized by toxic damage to liver cells or liver injury (Garcia-Cortes et al., 2020). The incidence of DILI has increased in recent years, now accounting for 3%–9% of adverse drug reactions globally (Saithanyamurthi and Faust, 2017). As DILI is predominantly a diagnosis exclusion, there is an urgent need for biomarkers of DILI. DILI has been posited to cause changes in exosomes, with detection of exosomes shown to have utility in the early diagnosis of DILI (Zhao et al., 2021b). In addition, biological molecules carried by exosomes can be delivered to nearby or distant cells, thereby altering their functions and affecting the progression of DILI (Usui and Naisbitt, 2017; Zhao et al., 2021b). Figure 2 and Table 1 show the roles of nucleic acids and proteins carried by exosomes in DILI.

**TABLE 1:** Function of nucleic acids and proteins carried by exosomes on drug-induced liver injury.

Types	Exosomal contents	Source of exosomes	Reference	Functions
	miR-455-3p	MSC	Shao et al.^47^	Reducing inflammation and liver damage
	miR-223	MSC	Chen et al.^48^	Exerting the liver protective effect
	miR-let-7i-5p	MSC	Chang et al.^49^	Ameliorating DILI
	miR-122	Hepatocyte	Holman et al.^43^	Evaluating drug hepatotoxicity
			Starckx et al.^44^	
		miR-122	Serum/Plasma	Bakshi et al.^51^	Ameliorating DILI
		miR-192		Cho et al.^52^	Predicting the drug hepatotoxicity
		miRNAs-122a-5p	Serum	Motawi et al.^53^	Biomarkers for liver damage
	Nucleic acids	miRNAs-192-5p			
miRNAs-193a-3p					
miR-296		Urine	Yang et al.^55^	Biomarkers for diagnosing DILI
miR-484				
miR-434				
miR-664				
miR-20b-3p				
miR-34c				
miR-330				
miR-185				
miR-291a-5p				
miR-433				
catecholamine-methyl transferase		Hepatocyte	Palomo et al.^45^	Biomarkers for diagnosing DILI
arginase 1				
CYP2d1				
HSP90 hpsa5				
fibronectin				
fibrinogens				
integrin 1b				
integrin-linked kinase				
					CD81 angio-poietin-like 4
					Rap1a
Proteins	CES3	Hepatocyte	Eva et al.^46^	Evaluating the liver injury or aggravating DILI
	SLC27A2			
	HSP90			
	HSP70			
	FRIL1			
	PrPc	Urine	Conde-Vancells et al.^54^	Biomarkers for diagnosing DILI
	CD26			
	Slc3a1			
	CD81			
					CD10

Note: DILI, drug-induced liver injury; MSC, mesenchymal stem cell.

### Cell-derived exosomes

Nucleic acids or proteins carried by exosomes may have utility as biomarkers for predicting DILI. Intrahepatic cell-derived exosomes are significantly changed during DILI. Holman et al. (2016) reported that miR-122 levels were significantly increased in hepatocyte-derived exosomes following acetaminophen treatment. Changes in miR-122 can be detected earlier than traditional liver injury markers, allowing early detection of drug hepatotoxicity (Starckx et al., 2013). In a rat model of DILI, proteomic analysis of hepatocyte-derived exosomes revealed increased levels of enzymes associated with liver injury (catecholamine-methyltransferase, arginase 1, and CYP2d1) and translation-related proteins (Hsp90 and Hpsa5) and decreased levels of apoptosis-regulating proteins (fibronectin, fibrinogen, integrin 1b, integrin-linked kinase, CD81, angiopoietin-like 4, and the RAS-associated protein, Rap1a) (Palomo et al., 2018). A separate study demonstrated increased expression levels of CES3, SLC27A2, HSP90, HSP70, and FRIL1 in hepatocyte-derived exosomes from rats treated with galactosamine (Rodriguez-Suarez et al., 2014).

Nucleic acids or proteins carried by exosomes may alleviate or aggravate DILI. Mesenchymal stem cells (MSCs) are important sources of exosomes. Shao et al. (2020) developed a mouse model of toxin-induced acute liver injury and demonstrated that miR-455-3p in human umbilical cord MSC-derived exosomes (hUC-MSC-exos) reduced the infiltration of macrophages and inflammatory factors, thereby alleviating liver injury. Bone marrow MSC-derived exosomes (BMSC-exos) with high expression of miR-223 markedly reversed S100 and LPS/ATP-induced liver injury by downregulating cytokines, NLRP3, and caspase-1, which further exerted a hepatoprotective effect (Chen et al., 2018). In addition, Chang et al. (2021a) found that miR-let-7i-5p in human placental chorionic MSC-derived exosomes (pcMSC-exos) inhibited apoptosis in hepatocytes, attenuated hepatic inflammatory responses, decreased liver injury scores, and ultimately improved liver injury. In summary, exosomes extracted from a range of intra- and extrahepatic cell types have important roles in DILI.

### Body fluid-derived exosomes

Body fluids are carriers of exosomes secreted by distant cell types, thereby allowing exosomes to exert paracrine effects. DILI can cause changes in body fluid-derived exosomes. Nucleic acids or proteins in body fluid-derived exosomes may have utility as biomarkers for predicting DILI. Several studies have demonstrated that miRNAs in humoral-derived exosomes can act as markers of liver injury and inflammation (Arrese et al., 2015). In serum-derived exosomes from patients with DILI induced by anti-tuberculosis drugs (isoniazid, rifampicin, pyrazinamide), miR-122 and miR-192 levels were substantially increased indicating their potential utility as predictors or therapeutic targets for liver damage caused by anti-tuberculosis drugs (Bakshi et al., 2021). A previous study reported that miR-122 and miR-192 were increased in plasma-derived exosomes from acetaminophen-induced DILI in rats, while the opposite results were obtained after adding N-acetylcysteine (Cho et al., 2017). Motawi et al. (2018) reported that serum exosomal miRNAs-122a-5p, 192-5p, and 193a-3p were associated with liver injury indicating their potential utility as markers of liver damage or in determining the etiology of liver injury. Notably, exosomal miRNA-122a-5p had stronger diagnostic performance.

Urine, as an excretory material, also contains abundant exosomes. Conde-Vancells et al., 2010 analyzed protein levels of urine-derived exosomes from a rat model of DILI and found that PrPc, Cd26, Slc3a1, Cd81, and Cd10 had utility as biomarkers for diagnosing DILI. Yang et al. (2012) identified ten urinary miRNAs (miR-296, miR-484, miR-434, miR-664, miR-20b-3p, miR-34c, miR-330, miR-185, miR-291a-5p, and miR-433) in rats treated with acetaminophen or carbon tetrachloride. These miRNAs may be transported in exosomes and urinary miRNAs may help distinguish liver injury due to hepatotoxic drugs from injury due to non-hepatotoxic causes. In addition, urine can be collected in bulk and this procedure is noninvasive, which is a unique advantage of diagnostic tests based on urine-derived exosomes. Taken together, these findings demonstrate that proteins or nucleic acids carried by humoral-derived exosomes may have utility as predictors of hepatotoxicity.

In summary, nucleic acids and proteins carried by exosomes from different sources are important players in DILI and may have utility in predicting the hepatotoxicity of drugs or as therapeutic targets for DILI.

## Exosomes and hepatic ischemia reperfusion injury

**FIGURE 3:**
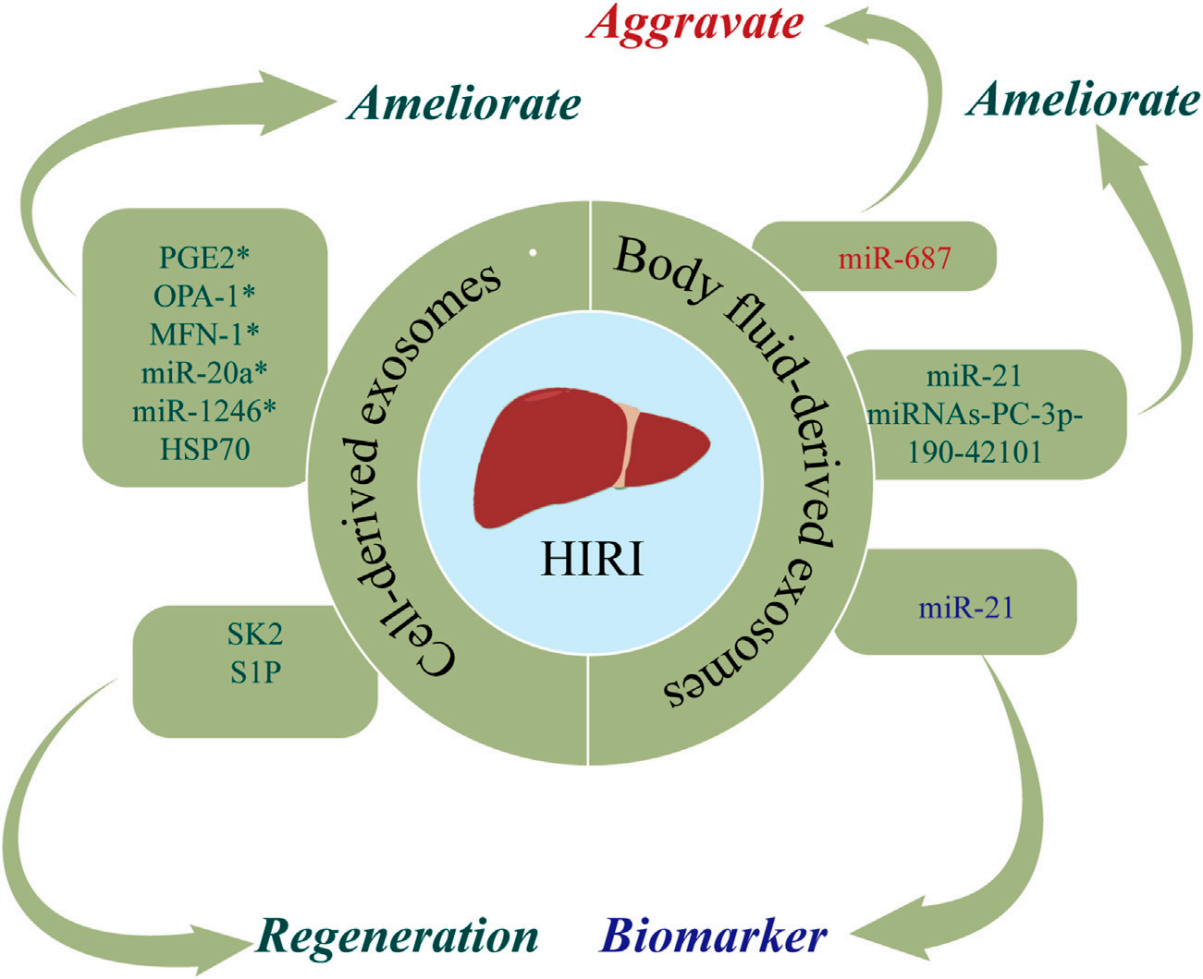
The roles of nucleic acids and proteins carried by exosomes in hepatic ischemia reperfusion injury (HIRI). Nucleic acids or proteins that may aggravate HIRI are highlighted in red, ameliorating HIRI are highlighted in green, and biomarkers are highlighted in purple. * refers to nucleic acids and proteins carried by exosomes from mesenchymal stem cell.

HIRI is a phenomenon in which hepatocytes are damaged due to transient ischemia, with liver damage further aggravated when blood flow is restored (Konishi and Lentsch, 2017). HIRI typically occurs after traumatic shock, liver surgery, or liver transplantation (LT) (Zhang et al., 2022a). At present, the most commonly used methods for preventing and treating HIRI are ischemic preconditioning (IPC), reducing the ischemia time, and inhibiting the inflammatory response after reperfusion (Wu et al., 2021c). However, due to the poor tolerance of liver tissue to hypoxia, there is an urgent clinical need for novel therapeutic strategies for HIRI. Exosomes are posited to exert protective and regenerative effects in HIRI (Zheng et al., 2018; Zhang et al., 2022b). Figure 3 and Table 2 show the roles of nucleic acids and proteins carried by exosomes in HIRI.

**TABLE 2:** Function of nucleic acids and proteins carried by exosomes on hepatic ischemia reperfusion injury.

Types	Exosomal contents	Source of exosomes	Reference	Functions
Nucleic acids	miR-1246	MSC	Xie et al.^63^	Alleviating HIRI
			Xie et al.^64^	
	miR-20a	MSC	Zhang et al.^65^	Alleviating HIRI
	miRNAs-PC-3p-190-42101	Plasma	Fei et al.^69^	Protect the liver from HIRI
	miR-687	Plasma	Ashour et al.^70^	Aggravating HIRI
	miR-21	Serum	Jia et al.^71^	Biomarkers for diagnosing
				HIRI or alleviating HIRI
Proteins	PGE2	MSC	Zhang et al.^60^	Alleviating HIRI
	OPA-1	MSC	Zhang et al.^66^	Ameliorating HIRI
	MFN-1			
	MFN-2			
	PGC-1α			
	NRF-1			
	TFAM			
	LC3-II	MSC	Yang et al.^67^	Alleviating HIRI
	SK2	MSC	Du et al.^68^	Accelerating liver
	SIP			regeneration
	HSP70	DC	Zheng et al.^59^	Alleviating HIRI

Note: HIRI, hepatic ischemia reperfusion injury; MSC, mesenchymal stem cell; DC: dendritic cell.

### Cell-derived exosomes

Nucleic acids or proteins carried by exosomes from different cells may alleviate HIRI. MSCs have emerged as a new therapeutic option for HIRI with effects including directed differentiation, induction of angiogenesis, tissue repair, and anti-inflammatory and anti-apoptotic activities (Phinney and Pittenger, 2017; Li et al., 2019). A previous study (Xie et al., 2019a) revealed that hUC-MSCs-exos regulated the GSK3β-mediated Wnt/β-catenin pathway by transporting miR-1246 after hypoxia/reoxygenation in LO2 cells, thereby inhibiting apoptosis and promoting cell proliferation to alleviate HIRI. Xie et al. (2019b) found that hUC-MSCs-exos could also regulate the balance between Tregs and Th17 cells through the miR-1246-mediated IL-6-gp130-STAT3 axis, thereby alleviating HIRI. Moreover, miR-20a in hUC-MSCs-exos can combine with the 3′UTR of Beclin-I and FAS to inhibit their expression, thereby ameliorating apoptosis in HIRI (Zhang et al., 2020a).

Adipose-derived mesenchymal stem cells (ADSCs) have latent effects on HIRI. Zhang et al. (2022b) confirmed that PGE2 in ADSC-derived exosomes (ADSCs-exos) mediated the phosphorylation of ERK1/2 and GSK-3β, upregulated Bcl-2, downregulated Bax, and reduced the levels of reactive oxygen species, thereby ameliorating inflammation, inhibiting apoptosis, and effectively protecting the liver from HIRI. A separate study (Zhang et al., 2021a) found that ADSCs-exos upregulated mitochondrial-associated proteins (OPA-1, MFN-1, MFN-2, PGC-1α, NRF-1, and TFAM), thereby maintaining mitochondrial homeostasis and ameliorating liver dysfunction in a rat model of HIRI. Yang et al. (2020) injected MSC-derived hepatocyte-like cell exosomes (MSC-Heps-exos) into the tail vein of HIRI mice and demonstrated increased levels of LC3-II, a marker of autophagy activity, increased hepatocyte autophagy, and decreased levels of circulating liver enzymes, thereby alleviating HIRI. Du et al. (2017) demonstrated that human-induced pluripotent stem cell-derived MSC-derived exosomes (hiPSC-MSCs-exos) could fuse with hepatocytes, thereby facilitating the synthesis of sphingosine kinase 2 (SK2) and sphingosine-1-phosphate (S1P), improving the tolerance of hepatocytes to hypoxia, increasing cell proliferation, and accelerating liver regeneration after HIRI. Moreover, they found that hepatocytes could activate the S1P pathway in the same way to promote cell proliferation. A further study (Zheng et al., 2018) confirmed that bone marrow-derived dendritic cell-derived exosomes (DEXs) transferred HSP70 into T cells and activated the PI3K/mTOR pathway to regulate the balance between Tregs and Th17 cells, ultimately having a protective effect on HIRI.

### Body fluid-derived exosomes

Nucleic acids or proteins in body fluid-derived exosomes may alleviate or aggravate HIRI. Fei et al. (2021) performed next-generation sequencing (NGS) of miRNAs in plasma-derived exosomes and verified that miRNAs-PC-3p-190-42101 in exosomes could reduce the levels of inflammatory factors and protect the liver from HIRI. In a rat model of HIRI, Ashour et al. (2021) reported that plasma exosomes had increased levels of miR-687, in addition to increased levels of liver tissue inflammatory markers and caspase-3. Inhibiting the expression of exosomal miR-687 may protect against hepatic injury. Therefore, exosomal miR-687 may play an important role in inducing HIRI. Furthermore, Jia et al. (2017) confirmed that serum exosomal miR-21 can inhibit the activity of NF-κb, downregulate programmed cell death protein 4, and upregulate bcl-2, thereby inhibiting apoptosis and reducing inflammation. Accordingly, serum exosomal miR-21 may represent a potential therapeutic target for HIRI.

Taken together, these results indicate that nucleic acids and proteins carried by exosomes play important roles in HIRI and may have utility in predicting HIRI. However, different molecules carried by exosomes may exert deleterious or protective effects on HIRI.

## Exosomes and hepatic fibrosis

**FIGURE 4:**
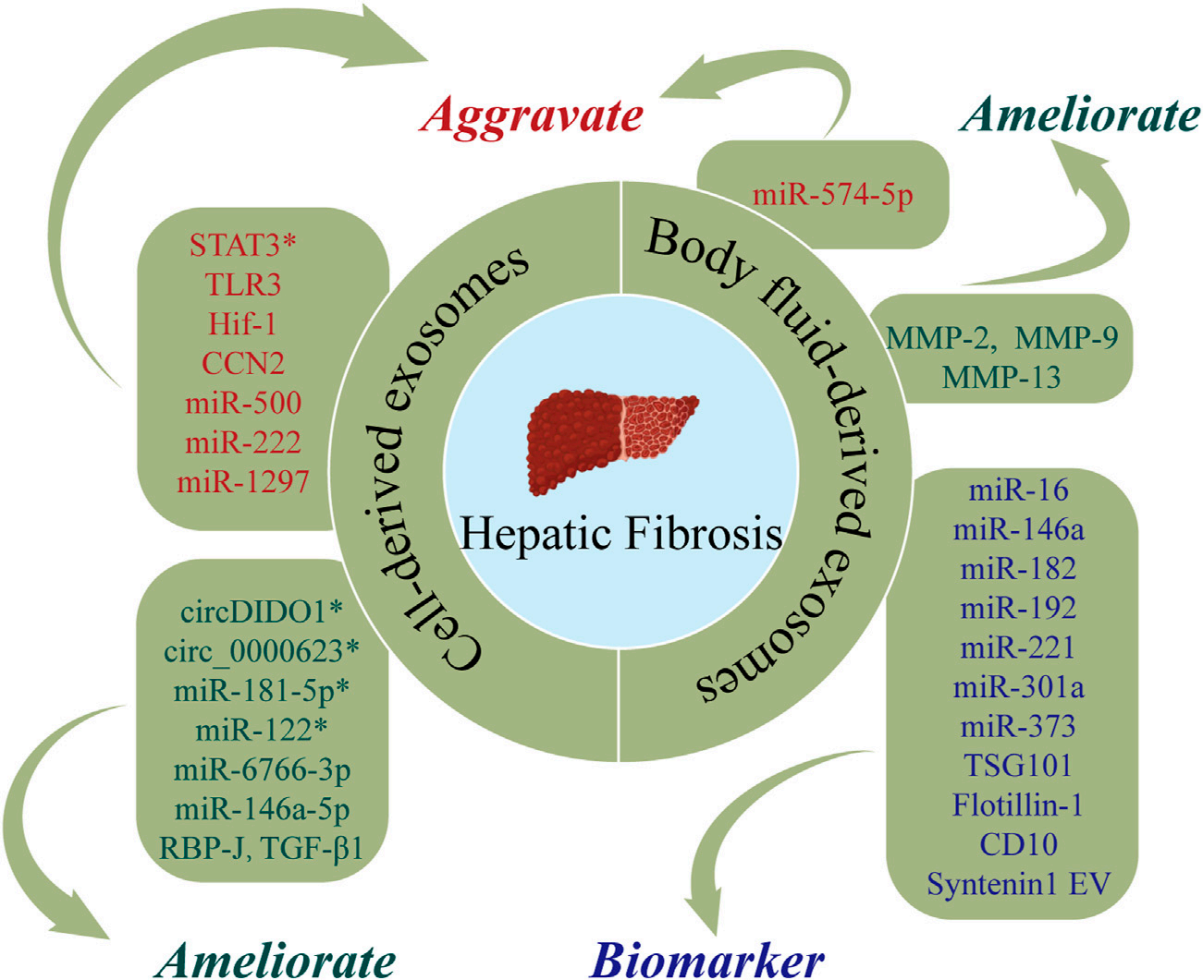
The roles of nucleic acids and proteins carried by exosomes in hepatic fibrosis (HF). Nucleic acids or proteins that may aggravate HF are highlighted in red, ameliorating HF are highlighted in green, and biomarkers are highlighted in purple. * refers to nucleic acids and proteins carried by exosomes from mesenchymal stem cell.

HF is a pathological process of excessive deposition and abnormal distribution of ECM after liver injury due to a range of etiologies (including alcohol, viruses, and autoimmune reactions) (Roehlen et al., 2020). The central link in HF is the activation of hepatic stellate cells (HSCs) (Sun and Kisseleva, 2015). HF may progress to cirrhosis that confers increased risks of liver cancer, LF, and death (Seki and Brenner, 2015). At present, there are no effective anti-fibrotic drug therapies for HF, with clinical treatment predominantly focusing on managing the underlying etiology and symptoms. Therefore, there is a need for studies of the effect of exosomes in HF. Figure 4 and Table 3 show the roles of nucleic acids and proteins carried by exosomes in HF.

**TABLE 3:** Function of nucleic acids and proteins carried by exosomes on hepatic fibrosis.

Types	Exosomal contents	Source of exosomes	Reference	Functions
Nucleic acids	circDIDO1	MSC	Ma et al.^75^	Alleviating HF
	mmu_circ_0000623	MSC	Zhu et al.^76^	Alleviating HF
	miR-181-5p	MSC	Qu et al.^77^	Alleviating HF
	miR-122	MSC	Lou et al.^78^	Alleviating HF
	miR-6766-3p	hESC	Wang et al.^79^	Alleviating HF
	miR-500	Macrophage	Chen et al.^83^	Aggravating HF
	miR-146a-5p	HLSC	Chiabotto et al.^84^	Alleviating HF
	miR-222	Hepatocyte	Zhang et al.^86^	Aggravating HF
	miR-1297	Hepatocyte	Luo et al.^87^	Aggravating HF
	lncRNA-H19	Cholangiocyte	Li et al.^88^	Aggravating HF
			Liu et al.^89^	
	miR-574-5p	Serum	Zhou et al.^92^	Aggravating HF
	miR-122	Serum	Chang et al.^93^	Prognostic biomarker for HF
	circDIDO1	Serum	Ma et al.^75^	Alleviating HF
	lncRNA-H19	Serum	Xiao et al.^94^	Aggravating HF
	miR-16	Plasma	Frundt et al.^97^	Being diagnostic and prognostic markers for HF
	miR-146a			
	miR-192			
	miR-221			
	miR-182	Ascites	Muhammad et al.^98^	Prognostic biomarker for HF
	miR-301a			
	miR-373			
Proteins	RBP-J	HEK293T cell	He et al.^80^	Alleviating HF
	TGF-β1	NK cell	Wang et al.^81^	Alleviating HF
	STAT3	MSC	Tang et al.^82^	Aggravating HF
	TLR3	Hepatocyte	Seo et al.^85^	Aggravating HF
	Hif-1	HSC	Wan et al.^90^	Aggravating HF
	CCN2	HSC	Charrier et al.^91^	Aggravating HF
	MMP-2	Plasma	Huang et al.^95^	Alleviating HF
	MMP-9			
	MMP-13			
	TSG101	Urine	Gonzalez et al.^96^	Diagnostic biomarkers for HF
	Flotillin-1			
	CD10			
	Syntenin1 EV			

Note: HF, hepatic fibrosis; MSC, mesenchymal stem cell; hESC, human embryonic stem cell; HLSC, human liver stem cell; HEK293T, human embryonic kidney cell; NK, cell, natural killer cell; HSC, hepatic stellate cell.

### Cell-derived exosomes

Nucleic acids or proteins carried by exosomes from different cells may alleviate or aggravate HF. HSCs play important roles in the pathogenesis of HF. Ma et al. (2022) revealed that MSC-derived exosomes delivered circDIDO1 to HSCs, which lead to inhibition of HSC activation through the miR-141-3p/PTEN/AKT pathway, thereby alleviating HF. ADSC-exos modified with mmu_circ_0000623 ameliorated HF by promoting autophagy (Zhu et al., 2020). Similarly, miR-181-5p-modified ADSC-exos activated autophagy and downregulated Stat3 and Bcl-2 in HST-T6 cells, thereby preventing HF (Qu et al., 2017). Lou et al. (2017) reported that MiR-122-modified ADSC-exos inhibited HSC activation, reduced collagen deposition, and ameliorated HF. A further study (Wang et al., 2021b) revealed that miR-6766-3p in 3D cultured human embryonic stem cell-derived exosomes (3D-hESC-exos) repressed the SMAD pathway by downregulating TGFβRII, thereby inhibiting HSC activation and slowing the progression of HF. He et al. (2022) demonstrated that HEK293T-derived exosomes effectively inhibited the Notch pathway in macrophages by delivering the transcription factor, RBP-J, thereby attenuating HF.

Wang et al. (2020) isolated exosomes from NK-92MI cells (NK-exos) and demonstrated that NK-exos inhibited the proliferation and activation of HSCs by downregulating TGF-β1, which further alleviated HF in mice. One study (Tang et al., 2021) designed fibroblast-like MSC-derived exosomes to carry siRNA or antisense oligonucleotides (ASOs) targeting STAT3, demonstrating that iExosiRNA-STAT3 or iExo-mASO-STAT3 downregulated STAT3, reduced ECM deposition, and ameliorated HF. Chen et al. (2021) demonstrated that macrophage-derived exosomal miR-500 promoted the proliferation and activation of HSCs by targeting MFN2, thereby aggravating HF. Chiabotto et al. (2021) concluded that human liver stem cell-derived extracellular vesicles (HLSC-EVs) attenuated the activation of HSCs by delivering miR-146a-5p. Seo et al. (2016) reported that hepatocyte-derived exosomes mediated the activation of TLR3, which upregulated IL-17A and aggravated HF. A recent study found that exosomal miR-222 from HBV-infected hepatocytes accelerated HF by inhibiting the transferrin receptor (TFRC) and ferroptosis (Zhang et al., 2022c). Lipotoxic hepatocyte-derived exosomal miR-1297 can promote the activation of HSCs via activation of the PTEN/PI3K/AKT signaling pathway (Luo et al., 2021). In addition, Li *et al.* (Li et al., 2018) and Liu et al. (2019) demonstrated that cholangiocyte-derived exosomes promoted the activation of HSCs by delivering lncRNA-H19 and accelerating the progression of cholestatic HF. Wan et al. (2019) revealed that hypoxia-inducible factor 1 (HIF-1) in activated HSC-derived exosomes mediated the transmission of glycolysis-related proteins (GLUT1 and PKM2) and enhanced glycolysis, thereby exacerbating HF. Moreover, Charrier et al. (2014) confirmed that HSC-derived exosomes can also accelerate the activation of HSCs by transmitting connective tissue growth factor (CCN2).

### Body fluid-derived exosomes

Nucleic acids or proteins in body fluid-derived exosomes may alleviate or aggravate HF and serve as biomarkers for predicting HF. A previous study (Zhou et al., 2022) extracted serum exosomes from healthy adults and patients with liver cirrhosis and co-cultured exosomes with a human hepatic stellate cell line, LX-2. This study found higher levels of serum exosomal miR-574-5p in patients with liver cirrhosis. Furthermore, serum exosomal miR-574-5p levels have been shown to be positively correlated with collagen deposition and *α*-SMA in liver tissue of HF mice. Chang et al. (2021b) extracted serum exosomes from 71 patients with HF for NGS, finding that exosomal miR-122 was negatively correlated with the degree of HF and serum exosomal miR-122 could act as a noninvasive predictor for HF. Conversely, downregulation of miR-122 accelerated the progression of HF. Ma et al. (2022) revealed that circDIDO1 in serum exosomes was associated with HF. Xiao et al. (2019b) reported that serum exosomal lncRNA-H19 promoted HF via the S1PR2/SphK2 and let-7/HMGA2 pathways, indicating that serum exosomal lncRNA-H19 may represent a novel therapeutic target for cholestatic HF. A separate study (Huang et al., 2021) found that upregulation of matrix metalloproteinases (MMP-2, MMP-9, MMP-13) in human umbilical cord blood plasma-derived exosomes (hUCB-exos) inhibited the accumulation of ECM and the progression of HF. Gonzalez et al. (2021) performed proteomic analysis of urinary extracellular vesicles (uEVs) from normal adults and patients with liver cirrhosis and identified 1,304 proteins. The levels of 90 proteins (such as TSG101, flotillin-1, CD10, and syntenin 1) were significantly altered, and these proteins were proposed as potential diagnostic biomarkers for liver cirrhosis. Fründt et al. (2021) posited that plasma exosomal miR-16, miR-146a, miR-192, and miR-221 are promising diagnostic and prognostic markers for liver cirrhosis. A further study (Muhammad Yusuf et al., 2020) showed that levels of ascites-derived exosomal miR-182, miR-301a, and miR-373 were elevated in patients with liver cirrhosis, indicating that these miRNAs may have utility as biomarkers in patients with liver cirrhosis.

Taken together, these studies demonstrate that nucleic acids and proteins carried by exosomes from different sources may aggravate or alleviate HF in certain conditions and have utility as biomarkers for predicting HF.

## Exosomes and liver failure

**FIGURE 5:**
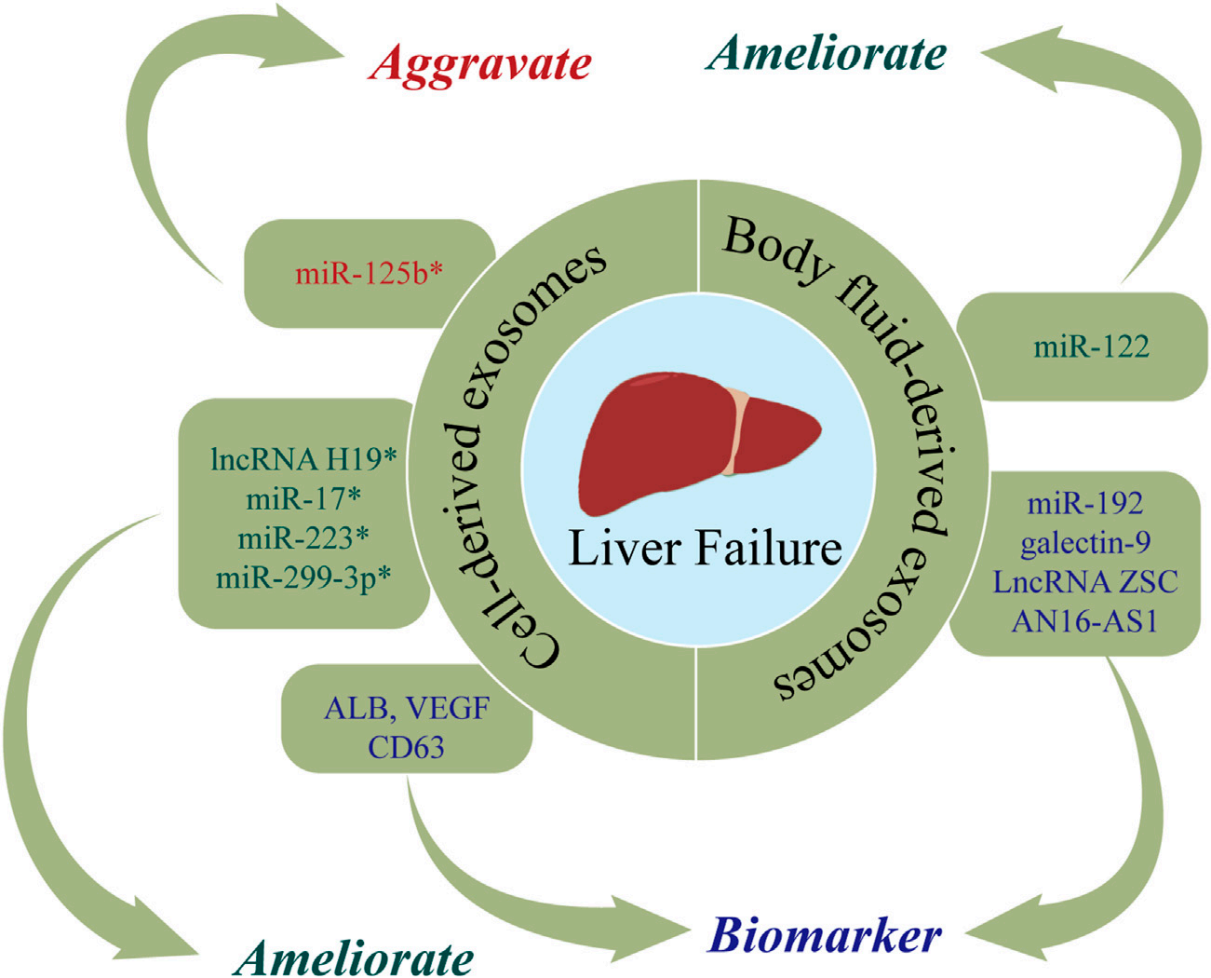
The roles of nucleic acids and proteins carried by exosomes in liver failure (LF). Nucleic acids or proteins that may aggravate LF are highlighted in red, ameliorating LF are highlighted in green, and biomarkers are highlighted in purple. * refers to nucleic acids and proteins carried by exosomes from mesenchymal stem cell.

LF is a clinical syndrome characterized by severe liver damage, coagulation disorders, jaundice, hepatic encephalopathy, and ascites (Liver, 2019). It has a high incidence, high mortality, and low cure rate (Putignano et al., 2018; Jalan et al., 2021). There are currently no specific pharmaceutical treatments for LF, with liver transplantation representing the only curative treatment option (Liver, 2019). However, LT is constrained by organ shortages, high costs, and the use of immunosuppressive drugs (Trebicka et al., 2020). Accordingly, there is an urgent need for novel treatments for LF. Figure 5 and Table 4 show the roles of nucleic acids and proteins carried by exosomes in LF.

**TABLE 4:** Function of nucleic acids and proteins carried by exosomes on liver failure.

Types	Exosomal contents	Source of exosomes	Reference	Functions
Nucleic acids	miR-17	MSC	Liu et al.^103^	Alleviating LF
	lncRNA H19	MSC	Jin et al.^104^	Alleviating LF
	miR-223	MSC	Chen et al.^48^	Alleviating LF
	miR-122	MSC	Lou et al.^78^	Alleviating LF
	miRNA-125b	MSC	Hyun et al.^107^	Aggravating LF
	miR-299-3p	MSC	Zhang et al.^108^	Alleviating LF
	miR-20a-5p	Hepatocyte	Zhang et al.^111^	Alleviating LF
	NOX1 mRNA	Serum	Chen et al.^113^	Biomarkers for predicting LF
	LncRNA ZSCAN16-AS1			
	miR-122	Plasma	Baker et al.^114^	Prognostic markers for predicting LF
	miR-192			
Proteins	GPX1	MSC	Yan et al.^105^	Alleviating LF
	ERK1/2	MSC	Wu et al.^106^	Alleviating LF
	PI3K			
	CRP	MSC	Jun et al.^109^	Alleviating LF
	ICAM-1 angiopoietin-2	MenSC	Chen et al.^110^	Alleviating LF
	Axl			
	Angiogenin			
	IGFBP-6			
	Osteoprotegerin			
	IL-6			
	IL-8			
	ALB	Hepatocyte	Jiao et al.^38^	Prognostic biomarkers for LF
	CD63			
	VEGF			
	Galectin-9	Plasma	Zhang et al.^112^	Being a prognostic marker for LF

Note: LF, liver failure; MSC, mesenchymal stem cell; MenSC, human menstrual blood stem cell.

### Cell-derived exosomes

Nucleic acids or proteins carried by exosomes from mesenchymal stem cells may alleviate or aggravate LF. Liu et al. (2018) found that ADSC-exos had therapeutic efficacy in ALF, while the effect was abolished after miR-17 knockout. MiR-17 ameliorated GalN/TNF-α-induced ALF by blocking the activation of NLRP3 in macrophages through targeting of TXNIP. Jin et al. (2018) injected exosomes derived from human adipose stem cells (hASCs) into ALF rats and observed that lncRNA H19 was upregulated, which in turn promoted the proliferation of hepatocytes and improved the survival rate of rats. The survival rate was reduced to 40% when lncRNA-H19 was silenced. A separate study (Yan et al., 2017) found that glutathione peroxidase 1 (GPX1) in hUC-MSCs-exos could counteract the toxic effects of CCl4 or H2O2, thereby reducing oxidative stress and exerting protective effects on LF. When GPX1 was knocked out, the protective effect was correspondingly attenuated. Wu et al. (2021d) found that hUC-MSCs-exos suppressed apoptosis and improved ALF by upregulating ERK1/2 and PI3K/AKT pathways. The opposite results were obtained with the addition of a PI3K or ERK1/2 inhibitor. Chen et al. (2018) infected BMSCs with pre-miR-223 and extracted BMSCs-exomiR-223 (+), demonstrating that BMSCs-exomiR-223 (+) decreased serum levels of ALT and AST, downregulated NLRP3 and caspase-1, and reversed liver injury. Furthermore, miR-122-modified ADSCs-exos attenuated collagen deposition by inhibiting the activation of HSCs (Lou et al., 2017). Hyun *et al.* (Hyun et al., 2015) revealed that inhibiting miRNA-125b in exosomes extracted from chorionic plate-derived MSCs (CP-MSCs) resulted in upregulation of Hh in HSCs, which in turn exacerbated LF. A further study (Zhang et al., 2020b) found that hUC-MSCs-exos inhibit activation of the NLRP3 pathway by delivering miR-299-3p, thereby reducing inflammation and promoting tissue repair. Jun et al. (2020) demonstrated that exosomal CRP from placental MSCs upregulated factors related to the Wnt pathway and angiogenesis in a rat model of LF, thereby promoting angiogenesis and liver regeneration. Chen et al. (2017) reported that exosomes extracted from human menstrual blood stem cells (MenSC-exos) inhibited apoptosis by promoting the expression of cytokines (ICAM-1, angiopoietin-2, Axl, angiogenin, IGFBP-6, osteoprotegerin, IL-6, and IL-8), and downregulating caspase-3.

Nucleic acids or proteins carried by exosomes from hepatocytes may alleviate LF and serve as important markers for predicting LF. Jiao et al. (2021b) extracted exosomes from hepatocytes of patients with acute-on-chronic LF (ACLF), demonstrating increased levels of ALB, CD63, and VEGF, which may represent more accurate prognostic indicators than alpha-fetoprotein (AFP). A separate study (Zhang et al., 2021b) indicated that miR-20a-5p was downregulated in hepatocyte-derived exosomes from ACLF mice, which in turn led to the upregulation of CXCL8 and increased inflammation. However, CXCL8 levels were decreased and liver injury was markedly alleviated after upregulation of exosomal miR-20a-5p.

### Body fluid-derived exosomes

Nucleic acids or proteins in body fluid-derived exosomes may have utility as markers for predicting LF. Zhang et al. (2019) revealed that plasma levels of exosomal galectin-9 in LF patients with acute cellular rejection were associated with poor prognosis, indicating that galectin-9 may be a predictor of rejection after liver transplantation. Chen et al. (2020) performed RNA sequencing of serum exosomes from normal adults and patients with ACLF caused by HBV (HBV-ACLF). They found that NOX1 mRNA and LncRNA ZSCAN16-AS1 were upregulated, indicating their potential utility as predictors for HBV-ACLF. Furthermore, Baker et al. (2015) reported that plasma exosomal miR-122 and miR-192 levels were increased at the onset of ALF, indicating their potential efficacy in predicting LF.

In conclusion, nucleic acids and proteins carried by exosomes from different cells may alleviate or aggravate LF and serve as important markers for predicting LF.

## Exosomes and liver cancer

**FIGURE 6:**
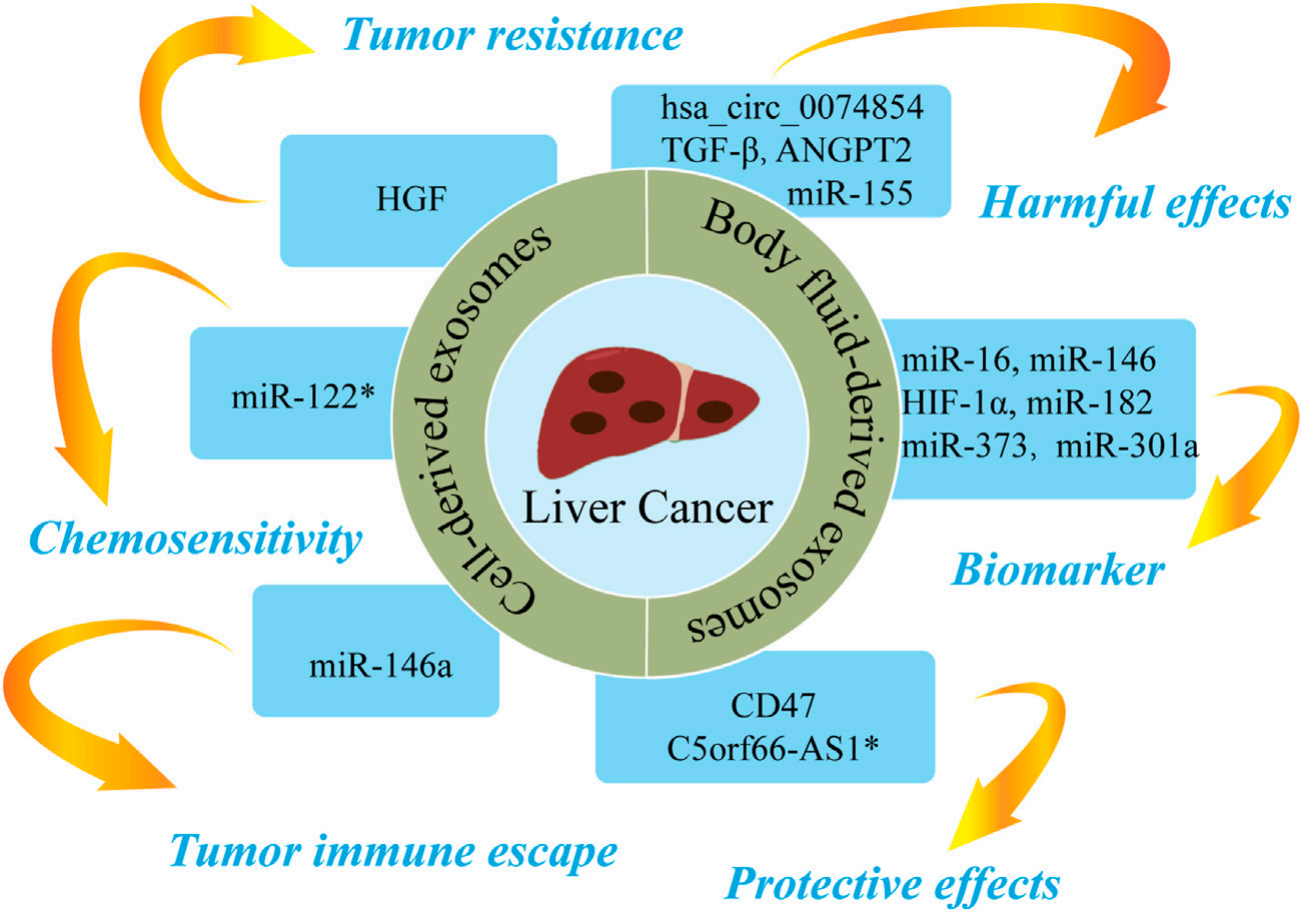
The roles of nucleic acids and proteins carried by exosomes in liver cancer. Nucleic acids or proteins carried by exosomes can exert harmful/protective effects on liver cancer and may also be predictive biomarkers for liver cancer. Some nucleic acids or proteins may be involved in tumor resistance, tumor immune escape, or chemosensitivity. * refers to nucleic acids and proteins carried by exosomes from mesenchymal stem cell.

Hepatocellular carcinoma (HCC) accounts for 75–85% of all liver cancers (Bray et al., 2018). It typically has an insidious onset, rapid development, and high mortality (Kulik and El-Serag, 2019). More than 800,000 people die from HCC each year, with a 5-years survival rate of approximately 6% (Raees et al., 2021). Therefore, there is an urgent clinical need for biomarkers that may contribute to the diagnosis and treatment of HCC. Increasing evidence indicates that the contents of exosomes are linked with tumor invasiveness and the tumor microenvironment and may influence the occurrence and development of HCC through related signaling pathways (An et al., 2018; Hwang and Yang, 2021). Figure 6 and Table 5 show the roles of nucleic acids and proteins carried by exosomes in liver cancer.

**TABLE 5:** Function of nucleic acids and proteins carried by exosomes on liver cancer.

Types	Exosomal contents	Source of exosomes	Reference	Functions
Nucleic acids	miRNA-210	Cancer cell/serum	Lin et al.^121^	Promoting cancer
	miR-155	Cancer cell/plasma	Matsuura et al.^122^	Promoting cancer
	miR-146a	Cancer cell	Han et al.^130^	Driving tumor immune escape
	hsa_circ_0074854	Cancer cell	Wang et al.^131^	Promoting cancer
	miR-660-5p	Macrophage	Tian et al.^132^	Promoting cancer
	miR-27a-3p	Macrophage	Li et al.^133^	Promoting cancer
	C5orf66-AS1	MSC	Gu et al.^134^	Suppressing cancer
	miR-122	MSC	Lou et al.^135^	Enhancing sensitivity to chemotherapy drugs
	miR-12	Serum	Mjelle et al.^138^	Prognostic predictors for liver cancer
	let-7 miRNA			
	miR-141			
	miR-146			
	miR-718	Serum	Sugimachi et al.^139^	Promoting cancer
	miR-16	Plasma	Frundt et al.^97^	Being diagnostic and prognostic biomarkers for liver cancer
	miR-146a			
	miR-192			
	miR-221			
	miR-182	Ascites	Muhammad et al.^98^	Being biomarkers for the diagnosis and prognosis of liver cancer
	miR-301a			
	miR-373			
Proteins	CLEC3B	Cancer cell	Xie et al.^123^	Promoting cancer
	HGF	Cancer cell	Qu et al.^124^	Promoting tumor resistance
	TGF-β	Cancer cell	Qu et al.^125^	Promoting cancer
	ANGPT2	Cancer cell	Xie et al.^126^	Promoting cancer
	AKT	Cancer cell	Wang et al.^127^	Promoting cancer
	STAT5α			
	ERK1/2			
	GSK3β			
	Shh	Cancer cell/plasma	Li et al.^128^	Promoting cancer
	S100A4	Cancer cell/plasma	Sun et al.^129^	Promoting cancer
	CD11b/CD18	Macrophage	Wu et al.^25^	Promoting cancer
	CD47	HEK293T cell	Du et al.^136^	Suppressing cancer
	AFP	DC	Lu et al.^137^	Suppressing cancer
	HIF-1α	Serum	Xu et al.^140^	Promoting cancer
	CPE	Serum	Hareendran et al.^141^	Promoting cancer
	TGF-β1	Ascites	Wei et al.^142^	Promoting cancer

Note: MSC, mesenchymal stem cell; HEK293T, human embryonic kidney cell; DC, dendritic cell.

### Cell-derived exosomes

Nucleic acids or proteins carried by exosomes from different cells may aggravate or alleviate HCC. Tumor cells can regulate the function of endothelial cells by releasing exosomal contents, thereby increasing vascular permeability and promoting angiogenesis and tumor metastasis (Aslan et al., 2019). Lin *et al.* (Lin et al., 2018) confirmed that miRNA-210 in HCC cell-derived exosomes (HCC-exos) can be transmitted to endothelial cells to downregulate SMAD4 and STAT6, thereby promoting angiogenesis. A study (Matsuura et al., 2019) revealed that miR-155 in HCC-exos induces angiogenesis and promotes tumor recurrence under hypoxic conditions. A further study (Xie et al., 2020a) demonstrated that CLEC3B in HCC-exos could inhibit the AMPK pathway and upregulate VEGF, thereby promoting angiogenesis and tumor progression. Qu et al. (2016) concluded that HCC-exos increases tumor resistance by upregulating HGF, activating the c-Met/Akt pathway, and repressing apoptosis. HCC-exos can also promote epithelial-mesenchymal transition (EMT) through the TGF-β/Smad pathway, thereby accelerating tumor metastasis (Qu et al., 2019). Similarly, HCC-exos has been shown to transport ANGPT2 to human umbilical vein endothelial cells via endocytosis, thereby promoting EMT *via* the Tie2-independent pathway (Xie et al., 2020b). HCC-exos can also activate phosphokinases (AKT, STAT5α, ERK1/2, and GSK3β), which in turn activate the NF-κB pathway to accelerate angiogenesis and cell migration (Wang et al., 2018). Li et al. (2021a) reported that HCC-exos secrete Shh and thereby activate the Hedgehog pathway, which increase cancer stem cells (CSCs) and promote cell proliferation. Sun et al. (2021) confirmed that S100A4 in HCC-exos promoted metastasis by activating STAT3 phosphorylation and upregulating OPN. A further study (Han et al., 2019) confirmed that miR-146a in HCC-exos could promote polarization of M2 macrophages and inhibit T-cell functions, thereby driving tumor immune escape. Furthermore, HCC-exos can transfer hsa_circ_0074854 to macrophages, with downregulation of hsa_circ_0074854 shown to repress the polarization of M2 macrophages and cell migration (Wang et al., 2021c).

Moreover, a previous study (Wu et al., 2021b) found that M2 macrophage-derived exosomes (M2-exos) delivered CD11b/CD18 to activate MMP-9 pathway and promote tumor metastasis. MiR-660-5p-modified M2-exos downregulated KHF3 to promote EMT and the development of HCC (Tian et al., 2021). Li et al. (2021b) concluded that miR-27a-3p in M2-exos downregulated TXNIP, thereby enhancing the stemness, drug resistance, and invasiveness of HCC cells. A separate study (Gu et al., 2021) reported that MSC-derived exosomes upregulated C5orf66-AS1 to activate the miR-127-3p/DUSP1/ERK axis and inhibit the malignant behavior of CSCs in HCC. Furthermore, miR-122-modified ADSCs-exos enhanced the sensitivity of HCC cells to chemotherapeutic drugs by regulating miR-122 (Lou et al., 2015). Du et al. (2021) confirmed that CD47-modified HEK293T cell-derived exosomes can induce ferroptosis, which may represent a novel therapeutic target for HCC. A further study (Lu et al., 2017) revealed that AFP-enriched DEX elicited antitumor immune responses and remodeled the tumor microenvironment, a mechanism that may have utility in immunotherapy for HCC.

### Body fluid-derived exosomes

Nucleic acids or proteins in body fluid-derived exosomes can alleviate or aggravate HCC and act as biomarkers for predicting HCC. Mjelle et al. (2019) performed RNA sequencing on serum from HCC patients. High levels of miR-12, let-7 miRNA, miR-141, and miR-146 in serum exosomes were found to be associated with poor survival, indicating that these miRNAs may have utility as prognostic predictors for HCC. In addition, miR-210 in serum exosomes was associated with microvessel density in HCC tissues (Lin et al., 2018). Serum exosomal miR-718 has been shown to be associated to tumor aggressiveness (Sugimachi et al., 2015), and plasma exosomal miR-155 has been shown to be associated with HCC recurrence (Matsuura et al., 2019). High levels of plasma exosomal Shh are associated with later tumor stage, higher histological grade, and higher recurrence in HCC (Li et al., 2021a). A further study (Sun et al., 2021) proposed exosomal S100A4 as a novel target for HCC metastasis as high levels of S100A4 in plasma exosomes were found to be associated with poor prognosis. Xu et al. (2019) found that HIF-1α in serum exosomes from HCC patients activated the PI3K/AKT pathway, thereby promoting angiogenesis and cell proliferation. Hareendran et al. (2022) reported that serum exosomal CPE was increased in HCC patients and HCC-exos loaded with CPE-shRNA inhibited cell proliferation by downregulating cyclin D1 and c-MYC. A further study (Frundt et al., 2021) proposed plasma exosomal miR-16, miR-146a, miR-192, and miR-221 as potential diagnostic and prognostic indicators in HCC patients. Furthermore, Muhammad Yusuf et al. (2020) demonstrated that ascites-derived exosomal miR-182, miR-301a, and miR-373 were upregulated in HCC, which may have utility in the diagnosis and prognosis of HCC patients. Wei et al. (2017) confirmed that ascites-derived exosomal TGF-β1 from HCC patients can promote the transformation of mesothelial cells into carcinoma-associated fibroblasts, thereby promoting peritoneal metastasis.

Taken together, these findings indicate that nucleic acids and proteins carried by exosomes from different sources can not only predict the recurrence and metastasis of HCC but may also play deleterious or protective roles in HCC.

## Conclusion and perspectives

Nucleic acids and proteins carried by exosomes may represent promising biomarkers for the diagnosis and treatment of liver diseases by facilitating early diagnosis and prognostication. Interestingly, some nucleic acids or proteins were identified in more than one study. Table 6 summarizes studies identifying nucleic acids and proteins carried by exosomes.

**TABLE 6:** Overview of the roles of nucleic acids and proteins carried by exosomes in different liver diseases.

Types	Exosomal contents	Liver diseases and references	Functions
Nucleic acids	miR-122	DILI^43-44,51–52^	Evaluating drug hepatotoxicity or ameliorating DILI
		HF^78,93^	Acting as a non-invasive predictor for HF or ameliorating HF
		LF^78,114^	Predicting the prognosis of LF or ameliorating LF
		Liver cancer^135^	Enhancing the sensitivity of cancer cells to chemotherapeutic drugs
	miR-223	DILI^48^	Exerting the liver protective effect
		LF^48^	Reversing liver injury
	miR-192	DILI^51-52^	Predicting the drug hepatotoxicity
		HF^97^	Being diagnostic and prognostic biomarker
		LF^114^	Predicting and assessing prognosis
		Liver cancer^97^	Being diagnostic and prognostic biomarker
	lncRNA-H19	HF^88-89,94^	Accelerating the progression of cholestatic HF, being the diagnostic biomarker and potential therapeutic target for cholestatic HF.
		LF^104^	Improving the survival rate and being a potential therapeutic target for LF.
	miR-221	HF^97^	Being a diagnostic and prognostic biomarker
		Liver cancer^97^	Being a diagnostic and prognostic biomarker
	miR-146a	HF^84,97^	Being a diagnostic and prognostic biomarker, or alleviating HF
		Liver cancer^97^	Being a diagnostic and prognostic biomarker
	miR-20a	HIRI^65^	Exerting the liver protective effect
		LF^111^	Aggravated liver inflammation
Proteins	HSP90	DILI^45-46^	Evaluating the liver injury
	CD81	DILI^45,54^	Biomarkers for the diagnosis of liver injury
	HSP70	DILI^46^	Evaluating the liver injury
		HIRI^59^	Exerting the liver protective effect
	CD10	DILI^54^	Biomarkers for the diagnosis of liver injury
		HF^96^	Biomarkers for the diagnosis of HF
	ERK1/2	LF^106^	Improving APAP-induced LF
		Liver cancer^127^	Promoting tumor metastasis
	TGF-β	HF^81^	Aggravating HF
		Liver cancer^125,142^	Promoting tumor metastasis

Note: DILI, drug-induced liver injury; HIRI, hepatic ischemia reperfusion injury; HF: hepatic fibrosis; LF, liver failure.

Despite initial studies of the role of exosomes in liver diseases, the development of exosome-related therapies for liver disease remains at the stage of *in vitro* and animal experiments, with a substantial amount of further development required to translate this work into clinical practice. Several outstanding challenges and issues have yet to be resolved. First, methods for mass production, isolation, purification, and preservation of exosomes are still in development. Second, the most appropriate and efficacious source of exosomes remains unclear. Third, there is a lack of knowledge regarding the biogenesis, release, targets, and molecular mechanisms of exosomes in the liver. Fourth, the safety and efficacy of exosomes in the treatment of liver disease have yet to be demonstrated. Therefore, further studies are required to overcome the obstacles to the translation of exosome research into clinical practice. With further research and improved technology, exosomes may represent a future therapeutic option for patients with liver diseases.
